# Molecular characterization of a new SARS-CoV-2 recombinant cluster XAG identified in Brazil

**DOI:** 10.3389/fmed.2022.1008600

**Published:** 2022-09-28

**Authors:** Thaís de Souza Silva, Richard Steiner Salvato, Tatiana Schäffer Gregianini, Ighor Arantes Gomes, Elisa Cavalcante Pereira, Eneida de Oliveira, André Luiz de Menezes, Regina Bones Barcellos, Fernanda Marques Godinho, Irina Riediger, Maria do Carmo Debur, Cristina Mendes de Oliveira, Rodrigo Ribeiro-Rodrigues, Fabio Miyajima, Fernando Stehling Dias, Adriano Abbud, Rubens do Monte-Neto, Carlos Eduardo Calzavara-Silva, Marilda Mendonça Siqueira, Gabriel Luz Wallau, Paola Cristina Resende, Gabriel da Rocha Fernandes, Pedro Alves

**Affiliations:** ^1^Instituto René Rachou, Fundação Oswaldo Cruz, Belo Horizonte, Brazil; ^2^Laboratório Central de Saúde Pública do Estado do Rio Grande do Sul, Porto Alegre, Brazil; ^3^Instituto Oswaldo Cruz, Fundação Oswaldo Cruz, Rio de Janeiro, Brazil; ^4^Laboratório Municipal de Referência, Setor de Biologia Molecular, Belo Horizonte, Brazil; ^5^Centro de Desenvolvimento Científico e Tecnológico, Porto Alegre, Brazil; ^6^Laboratório Central de Saúde Pública do Estado do Paraná, Curitiba, Brazil; ^7^Dasa, Barueri, Brazil; ^8^Laboratório Central de Saúde Pública do Estado do Espírito Santo, Vitória, Brazil; ^9^Fiocruz Ceará, Fundação Oswaldo Cruz, Eusébio, Brazil; ^10^Instituto Adolfo Lutz, São Paulo, Brazil; ^11^Instituto Aggeu Magalhães, Fundação Oswaldo Cruz, Rio de Janeiro, Brazil

**Keywords:** SARS-CoV-2, recombinant, genomic surveillance, XAG, variants, omicron

## Abstract

Recombination events have been described in the Coronaviridae family. Since the beginning of the SARS-CoV-2 pandemic, a variable degree of selection pressure has acted upon the virus, generating new strains with increased fitness in terms of viral transmission and antibody scape. Most of the SC2 variants of concern (VOC) detected so far carry a combination of key amino acid changes and indels. Recombination may also reshuffle existing genetic profiles of distinct strains, potentially giving origin to recombinant strains with altered phenotypes. However, co-infection and recombination events are challenging to detect and require in-depth curation of assembled genomes and sequencing reds. Here, we present the molecular characterization of a new SARS-CoV-2 recombinant between BA.1.1 and BA.2.23 Omicron lineages identified in Brazil. We characterized four mutations that had not been previously described in any of the recombinants already identified worldwide and described the likely breaking points. Moreover, through phylogenetic analysis, we showed that the newly named XAG lineage groups in a highly supported monophyletic clade confirmed its common evolutionary history from parental Omicron lineages and other recombinants already described. These observations were only possible thanks to the joint effort of bioinformatics tools auxiliary in genomic surveillance and the manual curation of experienced personnel, demonstrating the importance of genetic, and bioinformatic knowledge in genomics.

## Introduction

Since the first detection of SARS-CoV-2 in Wuhan, China (1), many new viral strains/lineages emerged, carrying some differences from the initial virus. Over time, the evolution and fixation of mutations, especially in variants of concern (VOC), demonstrates that new strains have a high chance of continuing to emerge due to the changing selective pressure on the virus lineages associated with extensive transmission worldwide (2). Epidemiological surveillance is essential for the control of COVID-19. However, genomic surveillance is just as important (3). Genome analyzes can provide additional information for epidemiological surveillance, demonstrating outbreak dynamics in space and time, characterizing transmission, and allowing the identification of mutations that can lead to the emergence of new variants with the potential to impact public health and the epidemiology of COVID-19 (4). The effectiveness of molecular surveillance as a tool for monitoring pandemics is dependent on continuous and consistent sampling through time and space, rapid virus genome sequencing, and rapid reporting (5). Enhancing genomic surveillance and sequencing efforts across the globe is a valuable tool to detect and understand emerging variants (6), and genomics-based SARS-CoV-2 surveillance is a helpful tool for monitoring the current and future phases of the COVID-19 pandemic (7).

Since the beginning of the COVID-19 pandemic, five lineages have been considered VOCs by the WHO: Alpha (B.1.1.7), Beta (B.1.351), Gamma (P.1), Delta (B.1.617.2), and Omicron (B.1.1.529). The Omicron variant, initially identified in South Africa and Botswana on November 21, 2021, was considered by the WHO to be a VOC on November 26, 2021 (8). Shortly after its identification, Omicron showed great potential for dissemination, with a significant increase over the Delta variant, which, since its identification, was the variant with the highest frequency worldwide. Omicron has been classified into five sublineages: BA.1/BA.1.*, BA.2/BA.2.*, BA.3, BA.4, and BA.5. BA.2 is replacing BA.1 as the dominant subvariant in more countries over time (9). To date (June 2022), the circulation of the BA.2/BA.2.* sublineage currently represents 61.5% of the genomes sequenced in Brazil (10). Omicron has potential for dissemination and should be closely monitored due to the high number of mutations present in the genome (at least 32) that can increase infectivity and immune escape compared with the early wild-type lineage and the other VOCs (11–13).

With the circulation of different variants in the same place and at the same time, co-infections become possible, potentially leading to the emergence, and rise of new variants through viral recombination. Unfortunately, the number of co-infections is challenging to determine, mainly because genomic surveillance is suboptimal in most countries (14). Some studies in different countries have found values ranging from 0.06 to 4.0% of co-infection and seem to be underestimated (15–20). Some tools based on metagenomics and bioinformatics have been proposed to identify and evaluate co-infections (21–25). It is unclear whether co-infections can result in more severe disease. However, the dominance of one strain over another in a co-infection has already been observed in the same patient, which can be explained by the higher virulence of the dominant strain (22). Genome recombination is an important evolutionary mechanism for the emergence and re-emergence of human pathogens and a significant source of viral evolution (9). SARS-CoV-2 originated from recombination may have advantages for viral dispersion, immune evasion potential, and decrease in vaccine effectiveness, but little is known about it and, consequently, it highlights the importance of studying the recombinants (26). Coronaviruses (CoVs) are highly recombinogenic, unlike other viruses that have emerged in the past two decades (27). Recombination occurs when genetic material from two circulating lineages is combined within the host, giving rise to a viable descent lineage (28). While some SARS-CoV-2 lineages disappear, others can become dominant through the fixation of key mutations in the genome that allow improved adaptation of these viruses regarding transmissibility and faster dispersion (6). The first recombinant to be identified, named XA, was detected in the UK in samples collected between Oct 2020 and Jan 2021 and resulted from recombination involving the Alpha variant (29). An increase in the detection of recombination events occurred between Delta and Omicron variants (20, 23, 30, 31) that co-circulated between November 2021 and February 2022 (10) and between Omicron variant sublineages such as BA.1 and BA.2 (32).

The gold standard technique for identifying and classifying SARS-CoV-2 lineages is based primarily on partial or whole genome sequencing through New Generation Sequencing (NGS) (6). Reverse transcription Polymerase chain reaction (RT-PCR) assays have been used to identify specific characteristics that are unique in specific variants, such as S-gene target failure (SGTF) in the Alpha and Omicron variant, and genotyping assays have also been applied for this purpose (33). Sanger sequencing is also a methodology that allows monitoring SARS-CoV-2 variants with a rapid response (34–36). Recombination can be challenging to detect by classification methods, as the recombinant sequences have high similarity to their shared ancestor (28, 32). Bioinformatics tools are used to identify and classify variants from the results obtained by NGS. However, these tools must be used with caution for potential new recombinant lineage classification once more in-depth human intervention is usually required for correct recombinant identification. Otherwise, recombinant lineages may be unreported until a high prevalence is reached (37, 38).

The aim of this study was the molecular characterization of a new recombinant lineage from samples collected in Brazil between April and May 2022. We demonstrate that this lineage was not initially classified by the available tools, such as Pangolin (v4.0.6 at that time). We performed several complementary analyzes and showed that this new recombinant, now named XAG, is the result of the recombination between two sublineages of the Omicron VOC, BA.1.1 and BA.2.23.

## Materials and methods

### Sequencing, variant calling, characterization, and phylogeny

The whole genome sequencing of the samples was performed using the COVIDSeq Illumina test protocol adapted by the Fiocruz Genomic Network.^1^ The assembly and variant calling was done through the ViralFlow (39) workflow that performs the assembly of the genome according to the reference sequence and additional analysis. The molecular characterization was performed by aligning the sequences with a dataset of sequences (Supplementary Data Sheet 1) from previously detected recombinant lineages submitted to the EpiCoV database at GISAID (40) using MAFFT (41) and visualization of the mutations profiles found through the AliView program (42). The multiple alignments were used in IQ-Tree (43) for estimating the Maximum-Likelihood phylogeny. Three different phylogenetic trees were reconstructed: I—Using the complete genome of the XAG recombinant, other available recombinant lineages, and parental BA.1 and BA.2 lineages; II–Using two fragments of the XAG recombinant genome splitted at the likely breaking point. The first fragment consists of the beginning of the genome up to 6,515 nt position (considered the probable recombination breakpoint), with characteristics like Omicron BA.1.* and the second fragment after this position, the genomic section likely derived from Omicron BA.2.*. A dataset with representative sequences of the following lineages was used: Omicron BA.1, BA.2, BA.2.23, XF (Delta/Omicron BA.1), XL (Omicron BA.1/BA.2), XG (Omicron BA.1/BA.2), XN (Omicron BA.1/BA.2), XQ (Omicron BA.1/BA.2), XR (Omicron BA.1/BA.2), and XAG (Omicron BA.1/BA.2). This study was reviewed and approved by Research Ethics Committee involving human beings at Instituto René Rachou, Fundação Oswaldo Cruz, under license protocol number: 4,084,902 and CAAE (certificate of presentation for ethical appreciation): 31984720300005091.

## Results

### Identification and classification of sequences collected in Brazil with initial variant identification failure

**FIGURE 1:**
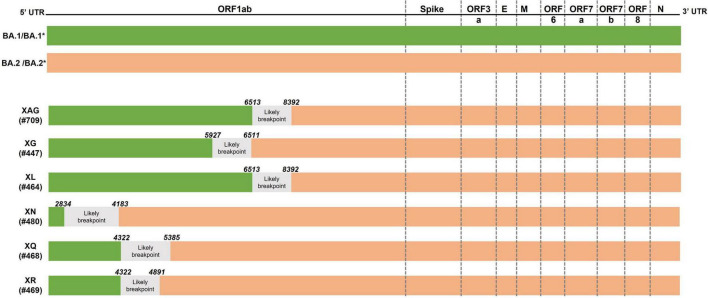
Schematic representation of recombination points. Schematic of the SARS-CoV-2 genome **(upper panel)** and positions where possible recombination points occur in the XAG, XG, XL, XN, XQ, and XR recombinants **(lower panel)**.

In May 2022, the first sequence (EPI_ISL_13019803) that failed to be identified by Pangolin was deposited and submitted to GISAID, identified in the Rio Grande do Sul, Brazil. The genetic profile present in this sample was detected in other submitted sequences collected later in Brazil. Through analysis of the mutations present in the sequences, a specific genetic signature of both the BA.1 and BA.2 lineages of the Omicron variant was identified, raising the hypothesis of recombination between these two lineages. In the genome analysis, a possible recombination point was detected between positions 6,512 and 8,395, at the same likely breaking point described for the XL recombinant. Up to position 6,512, the samples present a genetic signature characteristic of the BA.1.1 variant (including the deletion at position 6,512 found only in BA.1), while after position 8,392, the genetic signature resembles that of the BA.2.23 lineage (Figure 1 and Supplementary Table 1).

**FIGURE 2:**
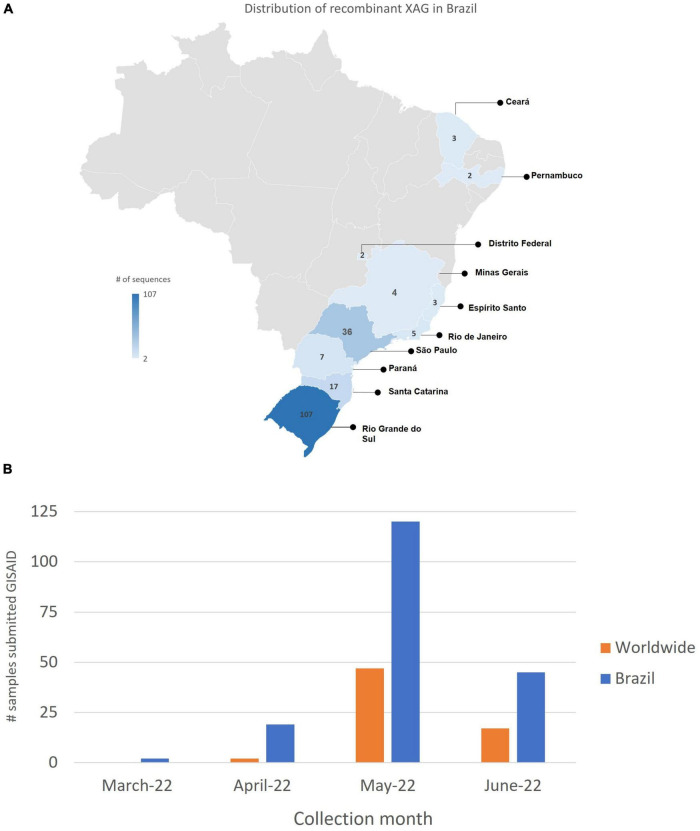
Detection of recombinant XAG in Brazil. **(A)** Until June 2022, 186 sequences were detected in 10 states of Brazil: 107 sequences in Rio Grande do Sul (RS), 7 in Paraná (PR), 17 in Santa Catarina (SC), 34 in São Paulo (SP), 3 in Espírito Santo (ES), 2 in Minas Gerais (MG), 5 in Rio de Janeiro (RJ), 2 in Distrito Federal (DF), 2 in Pernambuco (PE), and 3 in Ceará (CE). The XAG recombinant was detected in other countries such as Argentina, Austria, Canada, Chile, Colombia, Denmark, France, Germany, India, Israel, Japan, Luxembourg, Mexico, New Zealand, Peru, Portugal, Scotland, Switzerland, and USA. **(B)** The number of XAG sequences submitted to GISAID between March and June in Brazil and worldwide.

A new recombinant cluster was detected through molecular and phylogenetic analyses, later named XAG.^2^ The first sequence of the XAG variant was collected on March 10, 2022, in the city of Caxias do Sul, Rio Grande do Sul/Brazil, from a 17-year-old male patient. Currently, 186 sequences were detected in 10 Brazilian states (Figure 2) and have already been detected in other countries such as Argentina, Austria, Canada, Chile, Colombia, Denmark, France, Germany, India, Israel, Japan, Luxembourg, Mexico, New Zealand, Peru, Portugal, Scotland, Switzerland, and the USA. On July 7, 2022, 252 sequences belonging to the recombinant XAG cluster were deposited in GISAID (Supplementary Data Sheet 1).

### Phylogenetic analyzes and molecular characterization

**FIGURE 3:**
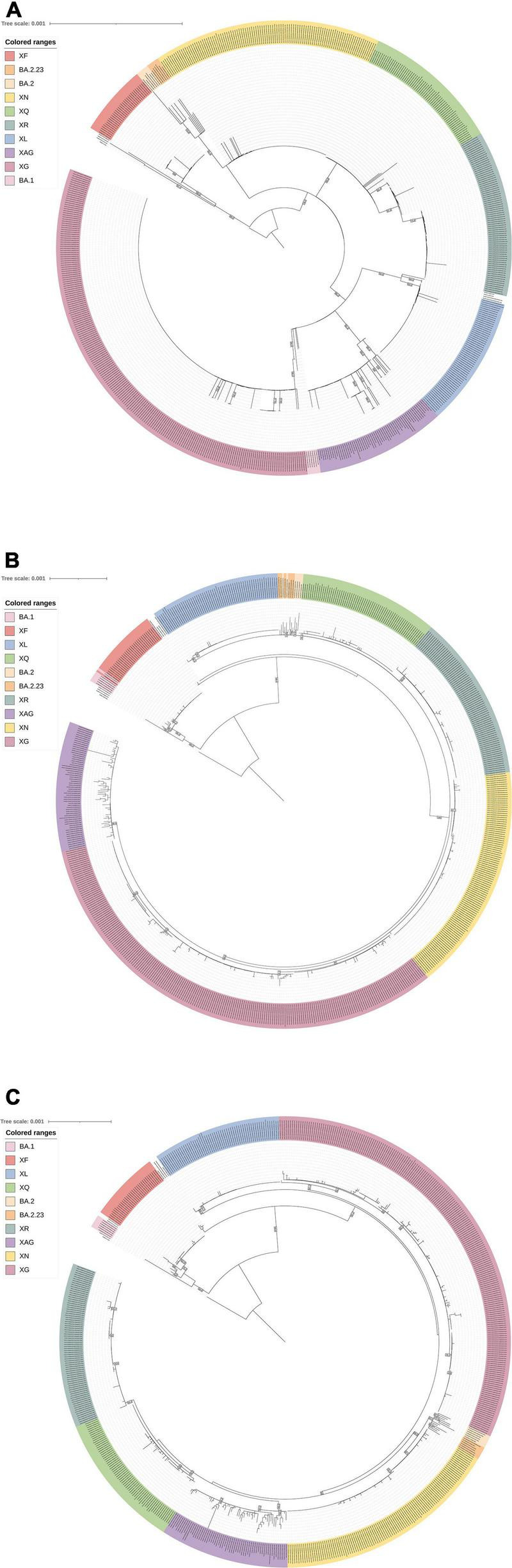
Phylogenetic analysis. The multiple alignment was used in IQ-Tree for estimating the Maximum-Likelihood phylogeny. Bootstrap analyzes were made using SH-like approximate likelihood ratio test with 1,000 replicates. **(A)** Phylogenetic tree reconstructed with the first fragment of the beginning of the genome up to 6,512 nt position (considered the probable recombination breakpoint), with characteristics like Omicron BA.1 (Model: TIM + F + I). **(B)** Phylogenetic tree reconstructed with the second fragment after the 6,512 nt position, the genomic section likely derived from Omicron BA.2/BA.2.23 (Model: TIM + F + R3). **(C)** Phylogenetic tree reconstructed with the complete genome of XAG genome, other available recombinant lineages and parental BA.1 and BA.2 lineages (Model: TIM + F + R3). A dataset with representative sequences of the following lineages was used: Omicron BA.1, BA.2, BA.2.23, XF (Delta/Omicron BA.1), XL (Omicron BA.1/BA.2), XG (Omicron BA.1/BA.2), XN (Omicron BA.1/BA.2), XQ (Omicron BA.1/BA.2), XR (Omicron BA.1/BA.2), and XAG (Omicron BA.1/BA.2).

In the phylogenetic analyses, it is possible to observe differences in the distances between BA.1 and BA.2 clusters when using the different genome portions. The proximity of the BA.1 branches to the BA.1-corresponding portion of the XAG genome is observed (Figure 3A), as well as the greater proximity of the BA.2/BA.2.23 branches to the portion corresponding to BA.2 of the XAG genome (Figure 3B). In the complete genome analysis of XAG, it is possible to verify that it is a new cluster, both regarding the recombinants of Omicron BA.1/BA.2 and XF (Delta/Omicron BA.1) (Figure 3C). Nevertheless, in less detailed analyses, it is possible to confuse the XAG recombinants with other Omicron BA.1/BA.2 recombinants such as XG and XQ.

**FIGURE 4:**
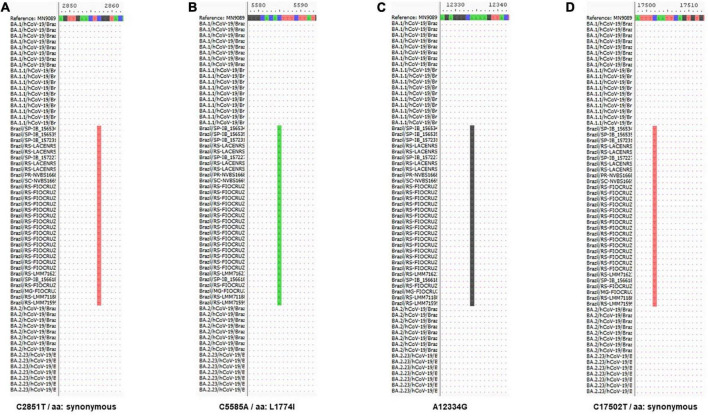
Molecular characterization of recombinant XAG. Alignment of sequences from Omicron subvariants BA.1 and BA.2, which originated the recombination that generated the XAG recombinant showed four distinct markers (in red) of this recombinant at positions **(A)** 2,857 (C/T), **(B)** 5,585 (C/A), **(C)** 12,334 (A/G), and **(D)** 17,502 (C/T). The mutation at positions 2,857 and 17,502 generate synonymous mutations and the mutation at position 5,585 (L1774I) is exclusive to this recombinant.

The XAG recombinant has four distinct markers: C2857T (synonymous), C5585A (L1774I), A12334G, and C17502T (synonymous), all present in the region of the ORF1ab gene. Up to position 6512, XAG has features of the BA.1 sublineage and, after that, shows a genetic signature like the BA.2 sublineage (Figure 4).

## Discussion

In nature, mutations, recombination, and reassortment are critical evolutionary processes that generate genetic diversity (44). The CoVs, like most other viruses, have developed a variety of genetic mechanisms, among which recombination and generation of defective-interfering (DI) RNA that, as a side effect, generate diversity (45, 46). SARS-CoV-2, despite being genetically distinct from the viruses that cause SARS epidemic in 2003 and Middle East respiratory syndrome coronavirus (MERS-CoV), shows high levels of genetic similarity with a strain from bats and strains obtained from pangolins (47–49), demonstrating that recombination can help to develop transmission strategies between species by establishing more susceptible hosts (50). Frequent genomes recombination, large genetic diversity, and high human-animal interface enable CoVs to emerge from time to time in humans due to occasional spillover and recurrent cross-species infectious events (44). Recombination and reassortment are essential processes that allow new antigenic combinations and altered phenotypes in emerging viruses that might aid the course of cross-species diffusion (44). RNA recombination is required during normal replication. The mechanisms and determinants of CoVs recombination are not known (51). Recombination of MERS-CoVs was already described in camels, leading to human outbreaks in 2015 (52). The high number of accumulated mutations in the Omicron variant may be due to recombination events. However, there is still no scientific evidence to support this hypothesis (53).

It was confirmed that co-infections by Omicron and Delta variants have already occurred in specific populations (9). Currently, the Omicron variant has the largest circulation in Brazil (approximately 99% of the genomes sequenced in June 2022), represented by 0.7% of the BA.1.* sublineage, 64% of the BA.2.*, 14.7% of the BA.4 and 20.5% of BA.5.* (10). This variant overlapped the Delta variant, but they co-circulated between November 2021 and January 2022, which contributed to co-infections and, consequently, allowed recombination between these two strains (9, 23). The first recombination identified was named XA and is the result of a recombination of the Alpha variant.^3^ After that, recombinant strains B.1.634 and B.1.631 were detected (XB), and so far, 32 recombinants have been identified in several countries,^4^ most of them occurred between Omicron variant sublineages recombination.

The XAG recombinant described in this work has four unique mutations. Two mutations, C2857T and C17502, are synonymous. Synonymous nucleotide changes may be related to virus adaptation and more efficient use of host tRNA profile, but it also may impact virus genome hairpins and 3D RNA structure (54). In addition, the mutation at position 5,585 (L17714I) is unique to the XAG recombinant and had already been observed in circulating strains previously, but at a low frequency, disappearing over time (55). SARS-CoV-2 and other CoVs have moderate genetic variability because they have an RNA-dependent RNA-polymerase (RdRP) with correction activity during viral genome replication and transcription (56). Even with this mechanism, errors can occur and become fixed if they present adaptive advantages. Host-related factors can also induce mutations, such as the antiviral mechanism mediated by APOBEC (Apolipoprotein B mRNA Editing Catalytic Polypeptide-like) proteins. APOBEC-like directional C→U transitions of genomic plus-strand RNA are overrepresented in SARS-CoV-2 genome sequences of variants emerging during the COVID-19 pandemic (57) and may affect the identification of co-infection events (15). De Maio et al. demonstrated that two mutation rates, C→U and G→U, are similar and much higher than all other mutation rates, leading to extremely frequent homoplasies (58). Sequence changes in SARS-CoV-2 and other coronavirus genomes may be partially or restricted to several mutational hot spots that promote convergent changes between otherwise genetically unlinked strains (59).

Recombination can be challenging to detect, mainly because they have similar characteristics to other circulating lineages at a higher frequency (9, 32). In addition to monitoring circulating lineages, the purpose of genomic surveillance is the rapid identification of new emerging lineages (5). Genomic surveillance also plays a significant role in studies on developing prophylactic measures and vaccines. Monitoring SARS-CoV-2 genetic changes, especially at the epitopes with implications for immune escape, is crucial (60). Since the first identification of a recombinant in Rio Grande do Sul, surveillance has been intensified, providing identification of more XAG recombinant sequences (see text footnote 1). This recombinant community transmission was observed in several neighboring and distant Brazilian states. This fact demonstrates the importance of genomic surveillance associated with an epidemiological link to validate genetic findings further.

As SARS-CoV-2 circulates worldwide, new lineages emerge and are tracked using the Pango dynamic hierarchical nomenclature system (28). Bioinformatics tools are essential and greatly help genomic surveillance, especially in pandemic scenarios, where surveillance needs to be assertive and fast (37). However, these tools have some limitations. In our work, it was possible to identify a flaw in the classification of the SARS-CoV-2 lineage by Pangolin since our sequences have mutations shared with the ancestral lineages and, in addition, they have synonymous mutations, which could only be detected through manual analyzes of all genome positions. To minimize this effect, Pangolin developers regularly train the tool with the latest designated sequencing (38). Despite this, viruses, especially RNA viruses, have high mutation rates that lead to an eminent environmental adaptation with rapid evolution, contrasting with the identification tools update time (44). We strongly suggest that classification tools are used allied with manual curation, especially in cases such as the one described in this study, since minority mutations may go unnoticed and help to tease apart co-infection and recombination events of epidemiological importance. The main limitation of our study is the focus only on the agent, based on the epidemiological triad (Agent, Host, and Environment), which demonstrates the need for further studies on recombinants (61, 62). We could not perform viral isolation, and it was also not possible to collect serial samples from each patient to assess the impact of XAG infection. The impact must be evaluated considering factors such as vaccination, social distancing measures, and recombination events that can occur in animals and viruses jumping back to humans (26, 61).

## Conclusion

In conclusion, it was possible to identify the emergence of a new SARS-CoV-2 recombinant, a result of the recombination between two sublineages of the VOC Omicron, applying bioinformatics tools for the identification of variants together with manual analyzes for the characterization of unique unlabeled mutations present in the new cluster, called XAG. In addition, new markers were identified in the XAG recombinant that had not been found in other previously described recombinants, demonstrating the potential for these viruses to evolve through recombination. This study demonstrates the need for continuous genomic surveillance of SARS-CoV-2, in which recombination appears essential in its evolution. The real impact of the recombinants needs to be further studied, considering the possibility of the occurrence of these events in animal-human interfaces and the emergence of new lineages.

## Data availability statement

The datasets presented in this study can be found in online repositories. The names of the repository/repositories and accession number(s) can be found in the article/Supplementary material.

## Ethics statement

The studies involving human participants were reviewed and approved by Research Ethics Committee involving human beings at Instituto René Rachou, Fundação Oswaldo Cruz, under license protocol number: 4084902 and CAAE (certificate of presentation for ethical appreciation): 31984720300005091. The ethics approval was issued on June 12, 2020. Written informed consent for participation was not required for this study in accordance with the national legislation and the institutional requirements.

## Author contributions

TS, PA, and GF: conceptualization, experimental design, and, writing—original draft. TS, PA, GF, RS, TG, IG, EP, RB, FG, IR, MD, CO, RR-R, FD, and AA: investigation and perform experiments. TS, PA, GF, GW, RS, TG, IG, EP, CO, FM, FD, AA, and PR: data analyses. RM-N, GF, PA, RS, TG, IG, EP, MS, and PR: reagents, materials, analysis, and tools. MS, GF, and PA: supervision. TS, PA, GF, RM-N, CC-S, RS, TG, IG, EP, MS, PR, EO, AM, RB, FG, IR, MD, CO, RR-R, FM, FD, AA, and GW: writing—review and editing. All authors discussed the results and contributed to the final manuscript.

## Footnotes

1
https://www.genomahcov.fiocruz.br/

2
https://github.com/cov-lineages/pango-designation/issues/709

3
https://virological.org/t/recombinant-sars-cov-2-genomes-involving-lineage-b-1-1-7-in-the-uk/658

4
https://github.com/cov-lineages/pango-designation
