# Supplementary material for: Molecular characterization of a new SARS-CoV-2 recombinant cluster XAG identified in Brazil
Source: Front Med (Lausanne). 2022 Sep 28;9:1008600. doi: 10.3389/fmed.2022.1008600 (PMC9554242; doi:10.3389/fmed.2022.1008600)
Supplement: Supplementary file 1 [file Data_Sheet_1.pdf]

SUPPLEMENTAL MATERIAL

Data Availability

GISAID Identifier: EPI_SET_220828ya
doi: 10.55876/gis8.220828ya

All genome sequences and associated metadata in this dataset are published in GISAID's EpiCoV database. To view the contributors of each individual sequence with details such as accession number, Virus name, Collection date, Originating Lab and Submitting Lab and the list of Authors, visit 10.55876/gis8.220828ya

Data Snapshot

- EPI_SET_220828ya is composed of 819 individual genome sequences.
- The collection dates range from 2021-03-09 to 2022-06-20;
- Data were collected in 21 countries and territories;
- All sequences in this dataset are compared relative to hCoV-19/Wuhan/WIV04/2019 (WIV04), the official reference sequence employed by GISAID (EPI_ISL_402124). Learn more at https://gisaid.org/WIV04.
