# Supplementary material for: Molecular characterization of a new SARS-CoV-2 recombinant cluster XAG identified in Brazil
Source: Front Med (Lausanne). 2022 Sep 28;9:1008600. doi: 10.3389/fmed.2022.1008600 (PMC9554242; doi:10.3389/fmed.2022.1008600)
Supplement: Supplementary file 2 [file Table_1.pdf]

Region	Aminoacid change	BA.1	BA.1.1	BA.2	BA.2.23	XAG
ORF1a	S135R	< 0,1%	< 0,1%	99,3%	< 0,1%	ND
	T842I	< 0,1%	< 0,1%	99,5%	97,6%	ND
	K856R	96,9%	96,7%	0,1%	1,9%	Present
	G1307S	< 0,1%	< 0,1%	99,8%	99,7%	ND
	L1774I *	ND	ND	ND	ND	Present
	S2083I	95,7%	95,5%	< 0,1%	< 0,1%	ND
	L2084I *	ND	ND	ND	ND	Present
	DEL2084/20284	95,7%	95,6%	< 0,1%	< 0,1%	ND
	A2710T	98,6%	99,0%	ND	ND	ND
	L3027F	< 0,1%	0,1%	99,0%	99,6%	Present
	T3090I	< 0,1%	0,1%	98,5%	99,4%	Present
	L3201F	< 0,1%	< 0,1%	96,4%	98,1%	Present
	T3255I	98,2%	99,0%	99,6%	99,6%	Present
	P3395H	97,6%	97,5%	99,8%	99,8%	Present
	DEL3674/3676	95,0%	95,4%	< 0,1%	< 0,1%	ND
	DEL3675/3677	< 0,1%	< 0,1%	97,6%	97,7%	ND
	I3758V	98,9%	99,5%	0,8%	0,2%	ND
ORF1b	P314L	98,6%	99,2%	99,8%	99,7%	Present
	R1315C	< 0,1%	0,2%	99,9%	99,7%	Present
	I1566V	93,7%	96,1%	97,9%	97,9%	Present
	T2163I	< 0,1%	0,1%	93,5%	90,0%	Present
SPIKE	T19L	< 0,1%	< 0,1%	98,8%	99,0%	ND
	L24S	< 0,1%	< 0,1%	94,8%	97,9%	ND
	A27S *	ND	ND	ND	ND	Present
	DEL25/27	< 0,1%	< 0,1%	94,9%	98,0%	ND
	A67V	96,4%	96,0%	< 0,1%	< 0,1%	ND
	DEL69/70	95,1%	95,0%	1,4%	0,1%	ND
	T95I	93,8%	94,9%	< 0,1%	< 0,1%	ND
	G142D	93,6%	92,8%	97,7%	99,3%	Present
	DEL143/145	93,6%	92,8%	< 0,1%	ND	ND
	N211I	84,0%	87,3%	0,1%	< 0,1%	ND
	DEL212/212	84,6%	87,5%	0,1%	< 0,1%	ND
	V213G	0,1%	< 0,1%	99,0%	99,6%	Present
	G339D	91,2%	93,4%	97,3%	99,0%	Present
	R346K	7,4%	92,3%	< 0,1%	< 0,1%	ND
	S371L	81,8%	87,8%	< 0,1%	< 0,1%	ND
	S371F	0,4%	0,1%	95,7%	98,1%	Present
	S373P	82,9%	88,4%	97,5%	99,3%	Present
	S375F	82,8%	88,2%	97,4%	99,3%	Present
	T376A	0,3%	< 0,1%	96,6%	99,2%	Present
	D405N	0,3%	< 0,1%	98,3%	99,7%	Present
	R408S	0,3%	< 0,1%	94,0%	95,3%	Present
	K417N	61,4%	70,4%	94,3%	97,8%	Present
	N440K	63,6%	74,9%	85,7%	96,9%	Present
	G446S	65,2%	76,2%	< 0,1%	< 0,1%	Present
	S477N	85,2%	91,0%	93,7%	99,2%	Present
	T478K	85,4%	91,1%	93,8%	99,2%	Present
	E484A	85,0%	91,2%	93,7%	99,2%	Present
	Q493R	84,6%	91,8%	92,7%	99,1%	Present
	G496S	81,3%	89,5%	< 0,1%	< 0,1%	ND
	Q498R	81,1%	89,2%	91,6%	98,9%	Present
	N501Y	81,6%	89,7%	91,9%	98,9%	Present
	Y505H	82,0%	89,3%	91,7%	98,9%	Present
	T547K	98,4%	99,1%	0,1%	< 0,1%	ND
	D614G	98,9%	99,4%	99,9%	99,7%	Present
	H655Y	98,6%	99,3%	99,8%	99,6%	Present
	N679K	98,6%	99,3%	99,8%	99,6%	Present
	P681H	98,2%	99,1%	99,4%	99,6%	Present
	N764K	86,4%	91,8%	97,1%	98,1%	Present
	D796Y	96,3%	97,8%	99,3%	99,1%	Present
	N856K	97,5%	98,7%	< 0,1%	ND	ND
	Q954H	97,5%	98,5%	99,7%	99,0%	Present
	N969K	97,9%	99,0%	99,7%	99,1%	Present
	L981F	97,7%	98,8%	ND	< 0,1%	ND
ORF3a	T223I	0,1%	0,2%	99,8%	99,7%	Present
E	T9I	98,1%	98,9%	99,2%	99,1%	Present
M	D3G	90,4%	92,3%	0,2%	ND	ND
	Q19E	94,2%	96,1%	93,6%	97,8%	Present
	A63T	96,1%	97,1%	99,3%	99,4%	Present
ORF6	D61L	< 0,1%	0,6%	96,4%	98,7%	Present
ORF8	S84L	95,8%	97,0%	97,7%	97,8%	ND
N	P13L	96,1%	97,0%	98,8%	98,3%	Present
	DEL31/33	93,6%	94,4%	96,0%	95,9%	ND
	R203K	96,5%	98,1%	97,0%	99,0%	Present
	G204R	96,7%	98,4%	97,1%	99,0%	Present
	S413R	< 0,1%	< 0,1%	98,0%	98,7%	Present
ORF9b	P10S *	ND	ND	ND	ND	Present

Supplementary table 1. Genetic signature of BA.1, BA.1.1, BA.2, BA.2.23 lineages and XAG recombinant. Values in parentheses represent mutation frequencies according to outbreak.info (45). ND: Not detected; * Mutations present only in recombinant XAG.
